# Total knee arthroplasty and bariatric surgery: change in BMI and risk of revision depending on sequence of surgery

**DOI:** 10.1186/s12893-023-01951-6

**Published:** 2023-03-10

**Authors:** Perna Ighani Arani, Per Wretenberg, Erik Stenberg, Johan Ottosson, Annette W-Dahl

**Affiliations:** 1 Department of Orthopedic Surgery, Örebro University Hospital, Örebro, Sweden; 2 Faculty of Medicine and Health, School of Medical Sciences, Örebro University, 702 81 Örebro, Sweden; 3 Department of Surgery, Örebro University Hospital, Örebro, Sweden; 4 Scandinavian Obesity Surgery Registry, Örebro, Sweden; 5 Department of Clinical Sciences Lund, Faculty of Medicine, Lund University, OrthopedicsLund, Sweden; 6 The Swedish Arthroplasty Register, Göteborg, Sweden

**Keywords:** Obesity, Bariatric surgery, Gonathrosis, Total knee arthroplasty, Revision

## Abstract

**Background:**

Patients with obesity have a higher risk of complications after total knee arthroplasty (TKA). We investigated the change in weight 1 and 2 years post-Bariatric Surgery (BS) in patients that had undergone both TKA and BS as well as the risk of revision after TKA based on if BS was performed before or after the TKA.

**Methods:**

Patients who had undergone BS within 2 years before or after TKA were identified from the Scandinavian Obesity Surgery Register (SOReg) and the Swedish Knee Arthroplasty Register (SKAR) between 2007 and 2019 and 2009 and 2020, respectively. The cohort was divided into two groups; patients who underwent TKA before BS (TKA-BS) and patients who underwent BS before TKA (BS-TKA). Multilinear regression analysis and a Cox proportional hazards model were used to analyze weight change after BS and the risk of revision after TKA.

**Results:**

Of the 584 patients included in the study, 119 patients underwent TKA before BS and 465 underwent BS before TKA. No association was detected between the sequence of surgery and total weight loss 1 and 2 years post-BS, − 0.1 (95% confidence interval (CI), − 1.7 to 1.5) and − 1.2 (95% CI, − 5.2 to 2.9), or the risk of revision after TKA [hazard ratio 1.54 (95% CI 0.5–4.5)].

**Conclusion:**

The sequence of surgery in patients undergoing both BS and TKA does not appear to be associated with weight loss after BS or the risk of revision after TKA.

## Background

Obesity is a global epidemic [1] and the prevalence has increased extensively over the past decades [2]. Obesity is also associated with dramatically increased morbidity and mortality [3] and is one of the most prominent risk factors for developing osteoarthritis (OA) [4]. The most effective method to counteract severe obesity, with its related comorbidities, is bariatric surgery (BS) [3].

Previous studies have described an increased overall risk of revision after total knee arthroplasty (TKA) in obese patients [5–7].

Both obesity and knee OA are prevalent conditions and as obesity rates continue to rise, the risk of developing knee OA also increases. As such, BS and TKA are important and potentially life-changing interventions, but it is important to determine the best sequence of action when both interventions are indicated. Given the difficulty of managing obesity, it would be beneficial to determine whether undergoing TKA prior to BS could aid in weight loss. Potentially, TKA prior to BS may facilitate improved postoperative rehabilitation and physical activity, thereby contributing to superior weight outcomes for patients. Additionally, performing BS prior to TKA could be hypothesized to reduce the risk of revision after TKA, given that obesity is associated with an increased risk of revision. Therefore, the aim of this study is to investigate if the sequence of surgery affects weight loss, measured 1 and 2 years post-BS, as well as the risk for revision after TKA.

## Methods

The Scandinavian Obesity Surgery Register (SOReg) was used to identify patients who underwent BS, defined as gastric bypass or sleeve gastrectomy, between 2007 and 2019. These patients were linked to the Swedish Knee Arthroplasty Register (SKAR) using patients' personal identification number (PIN), which are unique to every citizen in Sweden. Patients were eligible for inclusion if they had undergone a primary TKA due to OA between 2009–2020 and BS within 2 years before (BS-TKA) or after their TKA (TKA-BS).

Body Mass Index (BMI), age, the American Society of Anesthesiologists (ASA) classification at the time of TKA, information pertaining to later revisions, date of death, and emigration status were obtained from SKAR. BMI (height and weight), age, sex, type of surgery, and weight 1 and 2 years post-BS were obtained from SOReg. Patients with a missing BMI prior to BS or TKA were excluded. In patients who had staged bilateral TKA, with both being performed prior to the BS, the second TKA was included. In patients who had undergone staged bilateral TKA with both TKAs being performed after the BS, the first TKA was included.

When analyzing the change in weight, patients who had undergone TKA on both knees, where one was performed before the BS and the other one was performed after the BS, were only included in the BS-TKA group, as their knee OA could not be considered definitively treated until the second TKA was performed. When analyzing the risk of revision, both TKAs were included.

The outcome measures for this study were weight change after BS and revision after TKA. Weight change, assessed 1 and 2 years post-BS, was evaluated using the following parameters: change in BMI, total weight loss (TWL), and excess BMI loss (EBMIL). Additionally, revision was defined as a surgical procedure performed for any reason on an already resurfaced knee, where one or more of the components are exchanged, removed, or added, including arthrodesis and amputation.

### Statistics

The patients were divided into two groups depending on if they underwent TKA before or after BS: TKA-BS and BS-TKA. When comparing demographics and clinical characteristics between the cohorts; categorical variables were reported as counts and percentages while continuous variables were reported as means and standard deviations (SDs) or medians and interquartile ranges (IQRs). To evaluate the statistical significance of differences between the groups, Pearson’s Chi-squared test was used for categorical variables. For continuous variables, the Student’s t-test was used for normally distributed data, otherwise the Mann–Whitney U-test was applied.

The outcome measures of interest were weight change 1 and 2 years post-BS and revision after the primary TKA. In order to adjust for potential confounding, multilinear regression analysis was employed to determine the change in BMI, TWL (%), and EBMIL (%), based on the sequence of surgery. The regression models were adjusted for type of BS, sex, age and BMI at the time of BS. A Cox proportional hazards model was used to estimate the risk of revision for any reason and adjusting for sex, age, and BMI. Age and BMI at the time for the primary TKA were used in the adjustment in the Cox proportional hazards model.

Results of the multilinear regression models were reported as the average change in BMI, TQL, and EBMIL while the results of the Cox proportional hazards model were reported using a hazard ratio (HR). All values were presented with corresponding 95% confidence intervals (CI). Statistical significance was defined as a two-sided p value of less than 0.05. All analyses were performed using Statistical Package for the Social Sciences.

## Results

**Fig. 1:**
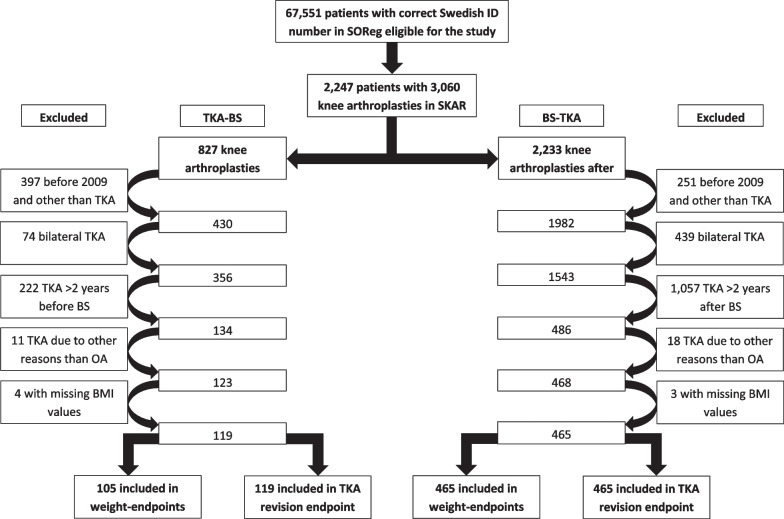
Flow chart of the study population

Of the 570 patients included in the analyses investigating the change in weight, 105 patients had undergone TKA for OA prior to BS and were included in the TKA-BS group while 465 patients underwent TKA for OA following BS and were included in the BS-TKA group (Fig. 1). The majority of the patients were women in both groups and the patients in the BS-TKA group were on average 2 years younger (Table 1). The median time between TKA and BS was 13 months in both groups.

**Table 1:** Patient characteristics at BS

	TKA-BS N = 105	BS-TKA N = 465	p value
Age in years, mean (SD)	57 (5.8)	55 (6.8)	0.015
Sex, n (%)			0.807
Female	80 (76%)	349 (75%)	
Male	25 (24%)	116 (25%)	
BMI, mean (SD)	40.5 (4.5)	43.1 (4.9)	< 0.001

*N* number; *SD* standard deviation; *BMI* Body Mass Index; *TKA* total knee arthroplasty; *BS* bariatric surgery

No statistically significant differences were detected in TWL or EBMIL 1 year post-BS, while an increased BMI-loss was observed in the BS-TKA group compared to the TKA-BS group [BMI-loss of 12.5 and 11.3 (Beta 1.3, 95% CI 0.4–2.1)]. However, the difference was no longer significant when adjusting for the potential confounders (Beta − 0.06, 95% CI − 0.8 to 0.6). 2 years post-BS, no statistically significant difference was found in BMI-loss, TWL or EBMIL (Table 2).

**Table 2:** Weight change after bariatric surgery depending on sequence of surgery

	BS-TKA		TKA-BS		Univariate regression		Adjusted regression	
	N	Mean ± SD	N	Mean ± SD	B (95% CI)	p-value	B^1^ (95% CI)	p-value
1 year								
BMI-loss	431	12.5 ± 3.9	96	11.3 ± 3.3	1.257 (0.414 to 2.1)	0.004	− 0.059 (− 0.760 to 0.642)	0.868
TWL, %	431	28.7 ± 7.5	96	27.6 ± 7.0	1.2 (− 0.5 to 2.8)	0.170	− 0.1 (− 1.7 to 1.5)	0.873
EBMIL, %	431	70.4 ± 19.4	95	74.6 ± 19.8	− 4.2 (− 8.5 to 0.1)	0.058	− 1.2 (− 5.2 to 2.9)	0.570
2 years								
BMI-loss	351	12.4 ± 4.4	75	11.4 ± 3.9	0.994 (− 0.095 to 2.082)	0.073	− 0.152 (− 1.038 to 0.734)	0.737
TWL, %	351	28.8 ± 8.9	75	28.0 ± 8.4	0.7 (− 1.5 to 2.9)	0.522	− 0.4 (− 2.5 to 1.6)	0.697
EBMIL, %	351	71.3 ± 22.4	74	76.0 ± 23.7	− 4.7 (− 10.4 to 1.0)	0.103	− 2.3 (− 7.5 to 3.0)	0.399

*N* number; *SD* standard deviation; *CI* confidence interval; *BMI* Body Mass Index; *TWL* total weight loss; *EBMIL* excess BMI-loss; *TKA* total knee arthroplasty; *BS* bariatric surgery

1 = Based on linear regression model, adjusted for age at bariatric surgery, sex, preoperative BMI and surgical method; Beta value for BS first compared to TKA first

The median weight change between TKA and BS in the TKA-BS group was a 5 kg (IQR 2–10) gain in weight and the median change in TWL was a 4.2% (IQR 1.6–9.7) increase in TWL. The mean weight change between BS and TKA in the BS-TKA group was a 33 kg (SD 13) loss in weight and the mean change in TWL was a 27% (SD 8) decrease in TWL.

When analyzing the risk of revision, 119 patients were included in the TKA-BS group and 465 patients were included in the BS-TKA group (Table 3). The median follow-up time was 39 months in the TKA-BS group and 24 months in the BS-TKA group. Five patients in the TKA-BS group (4.2%) underwent a TKA revision, while 26 patients in the BS-TKA group (5.6%) underwent a revision. No statistically significant difference in the risk of revision was detected when comparing the cohorts [HR 1.5 (95% CI 0.5–4.5)] (Table 4).

**Table 3:** Patient characteristics at TKA surgery

	TKA-BS N = 119	BS-TKA N = 465	p value
Age in years, mean (SD)	56 (5.7)	57 (6.8)	0.516
Sex, n (%)			0.749
Female	91 (76%)	349 (75%)	
Male	28 (24%)	116 (25%)	
ASA-classification, n (%)			0.12
1	10 (8%)	55 (12%)	
2	74 (62%)	312 (67%)	
≥ 3	35 (30%)	98 (21%)	
BMI, mean (SD)	38 (4.6)	31 (4.4)	< 0.001

*N* number; *SD* standard deviation; *TKA* total knee arthroplasty; *BS* Bariatric surgery; *ASA* American Society of Anaesthesiologists; *BMI* Body Mass Index

**Table 4:** Adjusted HR for the risk of revision after primary TKA

	N	HR (95% CI)	p-value
TKA-BS	119	Reference	Reference
BS-TKA	465	1.540 (0.526 to 4.509)	0.430

*HR* hazard ratio; *N* number; *CI* confidence interval; *TKA* total knee arthroplasty; *BS* bariatric surgery

## Discussion

This is the first study evaluating weight change after BS in patients who have undergone TKA either before or after BS. Furthermore, this is the largest study evaluating the association between the sequence of surgery and the risk of revision after TKA. The analyses were not able to identify an association between the sequence of surgery and weight change up to 2 years post-BS. Moreover, the sequence of surgery did not appear to be related to the risk of revision following TKA.

Previous studies investigating weight change after TKA have presented varying results [8–10]. Teichtal et al. observed 29 patients who had undergone TKA for 6 months postoperatively, with a mean BMI of 31.5 at the time of TKA. The majority of the patients (59%) lost weight (> 0 kg). When using 5% as a threshold for a clinically significant change in weight, 38% of the TKA patients had lost weight after the procedure. Furthermore, 35% of the patients had gained weight after the TKA (> 0 kg). The mean BMI change was − 1.07 (SD 1.80) corresponding to 3.3% (SD 5.7) reduction in weight [8]. In the current study, the median change of weight after TKA was found to be a gain of 5 kg in the TKA-BS group. Ast et al. reviewed 3,036 patients who underwent TKA with a mean BMI of 30.2 at the time of TKA. 2 years postoperatively, no change in BMI was seen in 69% of the patients [9]. In the current study, the patients in the TKA-BS group had a median weight gain of 4%. Inacio et al. assessed the change in weight before and after TKA or total hip arthroplasty (THA). They demonstrated that most of the patients who underwent TKA (68.5% of 20,060) exhibited an unchanged weight after the procedure, when defining a change of 5% as clinically significant. Nevertheless, these studies investigated patients with a lower BMI compared to our study, since all of the patients who underwent TKA prior to BS in our study where candidates for BS [10]. Nearing et al. (2017) evaluated the outcomes after TKA/THA in patients who had undergone BS either before or after their TKA/THA. This study included 102 patients who received a TKA/THA. TKA /THAs were performed at a mean of 4.9 years before and 4.3 years after BS. Obesity-related co-morbidities were similar between the two groups. Patients who underwent TKA/THA before BS demonstrated an average increase in BMI of 2.6 between the TKA/THA and BS, which is in line with our results. However, they found that patients who underwent TKA/THA after BS had a statistically significant lower BMI 1 year after the TKA/THA, compared to patients who underwent TKA/THA before BS [11]. However, in our study, we compared patients' weight 1-year post-BS instead of post-TKA. In a recently published randomized controlled trial analyzing the change in BMI and weight 1 year after TKA, the intervention group who received BS prior to TKA had a significantly greater BMI loss (− 6) and weight loss (− 16.5 kg) compared to the patients who underwent “treatment as usual” before TKA. However, 2/41 patients did not undergo BS prior to their TKA, and 12/41 did not undergo any TKA in the intervention group, but were still included in the intention to treat analysis. Furthermore, the majority of the patient in the intervention group underwent gastric banding which differs from the BS performed in the current study [12].

In a relatively recent systemic review, obesity was shown to increase the risk of revision following TKA [7]. Sezgin et al. found that obesity was associated with an increased overall risk of revision and revision due to infection, but could not show the same relationship for revision for reasons other than infection [13]. Since BS is an effective method of obtaining long-term weight loss [3], it is reasonable to believe that BS prior to TKA could reduce the risk of revision. However, in a previous study we did not find any association between a reduced risk of revision in patients undergoing BS prior to TKA [14]. Risk of revision based on the sequence of surgery has also been studied, demonstrating similar results to the current investigation [11, 15]. Nearing et al. (2017) did not detect any difference in the risk of revision, regardless of the timing of the TKA/THA in relation to the BS. The mean follow-up time after TKA/THA was 3.2 years in those who underwent TKA/THA after BS and 9.2 years in those who underwent TKA/THA before BS.  The current investigation was limited to a follow-up time of 2 years between the surgeries [11]. In a retrospective study, Kulkarni et al. (2011) evaluated the risk of revision after 1 year in 53 patients who underwent TKA/THA before BS and 90 patients who underwent TKA/THA after BS. No patients who underwent TKA, whether before or after BS, were reported to have required a revision within 1 year [15].

Despite these negative results, there are other factors that bear consideration. A recent retrospective cohort study investigated the risk of medical complications after the second operation in patients who underwent both BS and TKA/THA. When adjusting for comorbidities, their results indicated that BS before TKA/THA was associated with improved postoperative outcomes. However, they did not include revision of TKA in their outcomes [16].

Although the present study carries the benefits of using a nationwide cohort based on prospectively collected data from two high-quality sources [17, 18] it is not without limitations.

The current study is an observational study, thus it cannot making any claims about causality. Additionally, another significant limitation is the absence of data on comorbidities which were not available and thus not included in the analyses. Although the study utilizes the American Society of Anesthesiologists classification obtained from SKAR and the obesity surgery mortality risk score (OS-MRS) obtained from SOReg, these variables were not adjusted for in the regression model due to their interaction with BMI. [19, 20] Despite these limitations, it should be noted that the patients included in the study underwent elective surgeries and were optimized prior to the procedures by both the surgeon and anesthesiologist. The majority of the patients in both groups were classified as ASA 2 prior to the TKA. Additionally, to reduce the risk of confounding health factors affecting the outcome, the cut-off time between the two surgical procedures was set to 2 years. Finally, it is important to note that the 95% CIs are relatively wide, which may suggest a potential problem with achieving sufficient statistical power.

## Conclusion

The sequence of surgery in patients undergoing both BS and TKA does not appear to affect the weight loss after BS or the risk of revision after TKA. However, further study is required before changes can be made to relevant guidelines.

## Data Availability

Data cannot be shared publicly because of patient confidentiality under current Swedish legislation. Data are available from the Scandinavian Obesity Surgery Registry (contact via soreg@regionorebrolan.se), for researchers who meet the criteria for access to confidential data.

## Abbreviations

ASA: American Society of Anesthesiologists
BMI: Body Mass Index
BS: Bariatric surgery
CI: Confidence interval
EBMIL: Excess BMI loss
IQR: Interquartile range
OA: Osteoarthritis
OS-MRS: Obesity surgery mortality risk score
PIN: Personal identification number
SD: Standard deviation
SKAR: Swedish Knee Arthroplasty Register
SOReg: Scandinavian Obesity Surgery Register
THA: Total hip arthroplasty
TKA: Total knee arthroplasty
TWL: Total weight loss
